# Patients’ perception towards nursing care and its associated factors in adult inpatient departments in comprehensive specialized hospitals of Northwest Ethiopia, 2023: a mixed methods study

**DOI:** 10.1186/s12912-026-04988-z

**Published:** 2026-07-03

**Authors:** Aragaw Egziabherfenta Tadele, Alemshet Yirga Berhie, Dagmawit Zewdu, Asrat Yazew, Ayenew Genet Kebede, Yonas Wondie, Endalamaw Fentie Tadesse, Melaku Laikemariam, Bazezew Asfaw Guadie, Abebe Yenesew, Alemker Molla Yitayew, Gedfegn Shume Mogess, Yibeltal Addis Mekuria

**Affiliations:** 1 Department of Nursing, College of Medicine and Health Sciences, Injibara University, Injibara, Ethiopia; 2 Department of Adult Health Nursing, School of Health Sciences, College of Medicine and Health Sciences, Bahir Dar University, Bahir Dar, Ethiopia; 3 Department of Midwifery, College of Medicine and Health Sciences, Injibara University, Injibara, Ethiopia; 4 Department of Pediatrics and Child Health Nursing, College of Medicine and Health Sciences, Injibara University, Injibara, Ethiopia; 5 Department of Medical Laboratory Science, College of Medicine and Health Sciences, Injibara University, Injibara, Ethiopia; 6 Department of Biomedical Science, College of Medicine and Health Sciences, Injibara University, Injibara, Ethiopia; 7 Department of Environmental Health, College of Medicine and Health Sciences, Injibara University, Injibara, Ethiopia; 8 Department of Public Health, College of Medicine and Health Sciences, Injibara University, Injibara, Ethiopia

**Keywords:** Nursing care, Patient perception, Inpatient department, Associated factors

## Abstract

**Background:**

patients’ perceptions towards nursing care describes clients’ emotion about the nursing care they have received, which is used to provide essential and holistic information on the overall quality of nursing care. And there was no study that has been conducted in Ethiopia that explores patients’ perception towards nursing care through in–depth patient interviews incorporated with quantitative study. Therefore, this study has attempted to fill this gap.

**Methods:**

A convergent parallel mixed–methods design was carried out among 627 adult patients admitted to the inpatient departments of comprehensive, specialized hospitals in Northwest Ethiopia. From March 20, 2023, to April 27, 2023. Participants for the quantitative component were selected using systematic random sampling. For the qualitative component, the principal investigator purposively selected participants until data saturation. Variables with a p–value of less than 0.05 from the multivariable analysis were recognized as statistically significant, coupled with a 95% confidence interval. The Hosmer–Lemshow test was used to assess model fitness, and qualitative data were transcribed, coded, and thematically analyzed using ATLAS.ti version 8. Finally, results from both components were integrated during interpretation.

**Results:**

Out of the total 627 participants, 602 completed the study, yielding a response rate of 96.01%. Among the respondents, 50% had a good perception of nursing care. Multivariable analysis revealed that a good perception of nursing care was significantly associated with previous hospitalization history (AOR = 0.42; 95% CI: 0.29–0.63), admission to medical wards (AOR = 0.43; 95% CI: 0.27–0.69), and staying less than seven days in a hospital (AOR = 1.50; 95% CI: 1.03–2.20). In addition, qualitative findings identified key concerns, including communication gaps, delays in care provision, and variability in nurses’ attitudes.

**Conclusions:**

Approximately half of admitted adult patients reported a poor perception of nursing care. Prior hospitalization history, admission wards, and the length of time the patient spent in admission were factors associated with good perception of nursing care.

**Supplementary Information:**

The online version contains supplementary material available at https://doi.org/10.1186/s12912-026-04988-z.

## Background

Patients’ perception of nursing care refers to their subjective evaluation and feelings regarding the care provided by nurses during their hospitalization. It serves as a critical indicator of the quality and standards of healthcare services [1].

Because nurses interact with patients more frequently than any other healthcare professionals, they are the primary influencers of the overall patient experience [2, 3].

Understanding these perceptions is essential for healthcare organizations to identify gaps in care delivery and support quality improvement initiatives [4].

A positive perception is linked to better treatment adherence and trust, while a poor perception can lead to adverse outcomes, including patient dissatisfaction, delayed health–seeking behavior, and disengagement from care plans [5–10].

Globally, patients’ positive perception of nursing care shows wide variability, ranging from 18.5% to 94.1%, reflecting significant disparities across healthcare systems and socioeconomic settings [11–19]. In Africa, particularly in sub–Saharan regions, the documented rates of positive perception are notably lower, with negative perception rates reported between 18.5% and 50.3% [1, 5, 11, 12].

In Ethiopia, despite various government initiatives to improve inpatient services, suboptimal perception remains a persistent challenge [1, 5, 12]. Previous evidence suggests that these perceptions are influenced by a complex interplay of socio–demographic and clinical factors, such as age, sex, length of hospital stay, and the presence of chronic illness [13, 14, 20–28].

While several studies have assessed these perceptions in Ethiopia, there is a lack of evidence integrating quantitative assessments with in–depth qualitative insights to capture the nuance of the patient experience. Therefore, this study aims to assess patients’ perception of nursing care and its associated factors in adult inpatient departments of comprehensive specialized hospitals in Northwest Ethiopia using a mixed–methods approach.

## Methods

### Study design and setting

A convergent parallel mixed–methods design was conducted from March 20 to April 27, 2023, in adult inpatient departments of comprehensive specialized hospitals in Northwest Ethiopia. Among the five hospitals in the region, Felege Hiwot Comprehensive Specialized Hospital (FHCSH) and Tibebe Ghion Comprehensive Specialized Hospital (TGCSH) were randomly selected using a lottery method. Both hospitals are located in Bahir Dar, the capital city of the Amhara regional state, around 565 km from Addis Ababa, and serve an estimated 7 million people [21]. FHCSH has 424 nurses, while TGCSH has 310 nurses, and both provide a wide range of clinical services.

### Study population

The source population included all adult patients admitted to the inpatient units of comprehensive, specialized hospitals in Northwest Ethiopia. While study populations were patients who were admitted to the inpatient departments in selected comprehensive specialized hospitals in Northwest Ethiopia and who were admitted to surgical, medical, & orthopedic wards whose age was ≥ 18 years, those who stayed for greater than 24 h prior to the data collection time were included in the quantitative study, and for the qualitative component, participants who had been hospitalized for at least five days at the time of discharge were included in the interview.

### Sample size determination

The sample size for the quantitative component was calculated using a single population proportion formula, assuming a 95% confidence level, a 5% margin of error, and a prevalence of 45.2% [5]. After applying a design effect of 1.5 and adding a 10% non–response rate, the final sample size was 627.

For the qualitative study, participants were included in the in–depth interview based on the saturation level of the data.

### Sampling procedure

For the quantitative study, a multistage sampling technique was utilized to obtain a representative sample of patients from admitted patients. Initially, we prepared a list of hospitals that are found in northwest Ethiopia, such as Debremarkos Comprehensive Specialized Hospital, Felegehiwot Comprehensive Specialized Hospital, Debretabor Comprehensive Specialized Hospital, University of Gondar Comprehensive Specialized Hospital, and Tibebeghion Comprehensive Specialized Hospital. Using the simple random sampling method, two hospitals, such as Felegehiwot Comprehensive Specialized Hospital and Tibebeghion Comprehensive Specialized Hospitals, were selected. Wards (medical, surgical, and orthopedic) were used as strata, with sample sizes allocated proportionally based on previous patient flow. Then samples were accessed for data collection after proportional allocation to each hospital; in order to know the average monthly inpatient admissions to selected wards of both hospitals, last year’s monthly Inpatient Department (IPD) reports were taken and divided by twelve, and it yielded TGCSH = 682 & FHCSH = 587 & the summation of average monthly admissions for both hospitals was 1269, and we calculated the K–value as (1269/627 ≈ 2). Finally, we employed the lottery method to select the first patients from the admission registration log books of selected wards, and others were selected using systematic random sampling every 2nd patient. For the qualitative study, the principal investigator performed in–depth interviews with purposively selected participants until data saturation.

### Study variables

#### Dependent variable

Patients’ perceptionss of nursing care.

#### Independent variables

**Socio–demographic factors**: age, sex, educational status, marital status, occupation, residence & monthly income.

**Patient–related factors**: previous history of admission, duration of hospital stay, having a companion, and medical condition.

**Institution/hospital–related factors**: type of admission room, ward service type, unit of hospital & type of hospital admission.

### Operational definition

**Good perception**: Having a score above the median score of 26 item questions under perception [22].

**Poor perception**: Having a score less than or equal to the median score of questions under perception [1].

**Inpatient Department**: Provides services for admitted patients who were registered by liaison officers [27].

### Data collection procedures

Quantitative data were collected using a structured Likert–scale questionnaire (1–5) adopted from previous study [1]. Data collection was carried out by four trained BSc nurses under the supervision of one BSc nurse.

Qualitative data were collected through in–depth interviews conducted by the principal investigator in a quiet and convenient setting. Interviews were audio–recorded with consent, transcribed verbatim, and translated into English. Pseudonyms or codes were used to ensure confidentiality.

### Data quality assurance

To ensure high data quality, a series of systematic steps were undertaken. The questionnaire was initially developed in English, then translated into Amharic, followed by back–translation to English to verify consistency. Data collectors and supervisors were given one day of training on the study procedures before data collection began. A pretest was carried out on 5% of the total sample at the adult inpatient wards of the University of Gondar Comprehensive Specialized Hospital to assess the accuracy of the questionnaire. During the actual data collection process, supervisors and the principal investigator checked the data daily for completeness and consistency.

For the qualitative part of the study, the quality and trustworthiness of the data were preserved by focusing on credibility, dependability, transferability, and confirmability. This was achieved through spending sufficient time in the field, keeping detailed records, asking probing questions during interviews, and cross–checking the written transcripts with the original audio recordings.

### Data analysis

The quantitative and qualitative data were collected simultaneously and analyzed separately. Hence, quantitative data were systematically coded and entered into EpiData version 4.6, then exported to SPSS version 25 for further analysis, and binary logistic regression was performed. Variables with *p* < 0.25 in bivariable analysis were entered into the multivariable model, and the Hosmer–Lemeshow test was used to confirm a good model fit (*p* = 0.763). The findings are presented clearly through both narrative descriptions and tabular formats.

Whereas qualitative data were transcribed, translated, and analyzed thematically. Coding and categorization were supported using ATLAS.ti version 8.Finally, integration of quantitative and qualitative findings was conducted using a triangulation approach, where qualitative themes were used to explain and contextualize statistically significant quantitative results.

## Results

### Quantitative results

#### Sociodemographic characteristics of the respondents

In this study, 602 patients participated with the response rate of 96.01%. Among the respondents, 325 (54.3%) were males (Table 1).

**Table 1:** Socio–demographic characteristics of patients in comprehensive specialized hospitals of northwest Ethiopia, 2023

Variables	Category	Frequency(*n*)	Percentage (%)
Sex	Male	325	54
	Female	277	46
Age	18–30	220	36.5
	31–40	110	18.3
	41–50	113	18.8
	51 and above	159	26.4
Marital status	Single	165	27.4
	Married	335	55.6
	Separated	24	4
	Divorced	39	6.5
	Widowed	39	6.5
Educational status	Unable to read and write	200	33.2
	Able to read and write	92	15.3
	Primary school	118	19.6
	Secondary School	111	18.4
	College diploma and above	81	13.5
Occupation	Government employee	87	14.5
	Merchant	92	15.3
	Farmer	217	36
	House wife	65	10.8
	Student	64	10.6
	Private employee	52	8.6
	Others	25	4.2
Place of residence	Rural	333	55.3
	Urban	269	44.7
Monthly income	< 500	11	1.8
	500–1000	14	2.3
	1001–1500	36	6
	1501–2000	150	24.9
	> 2000	391	65

Others (Occupation): – Daily laborers, prisoners, religious fathers & retired

### Patient and admission related characteristics

In terms of patient and admission–related characteristics, the majority of study participants (41%) were admitted to the medical ward, whereas 60.8% of participants had spent > = 8 days in the study hospitals. Of the participants, 406 (67.4%) had never been previously admitted, and the majority of respondents (94.7%) reported having companionship during their hospital stay (Table 2).

**Table 2:** Patient and admission–related characteristics of the participants in comprehensive specialized hospitals of Northwest Ethiopia, 2023

Variable	Category	Frequency(*n*)	Percentage (%)
Ward	Medical	247	41
	Surgical	232	38.5
	Orthopedics	123	20.4
Number of days stayed in hospital	< 7	194	32.2
	> 8	408	67.8
Service type	Free	49	8.1
	Community Based Health Insurance (CBHI)	396	65.8
	Payment	157	26.1
History of previous admission	Yes	196	32.6
	No	406	67.4
Companion/Family support	Yes	570	94.7
	No	32	5.3
Medical Conditions	Acute illness	417	69.3
	Chronic illness	185	30.7
Type of hospital admission	Planned	77	12.8
	Un Planned	525	87.2

### Patients’ perception towards nursing care

**Fig. 1:**
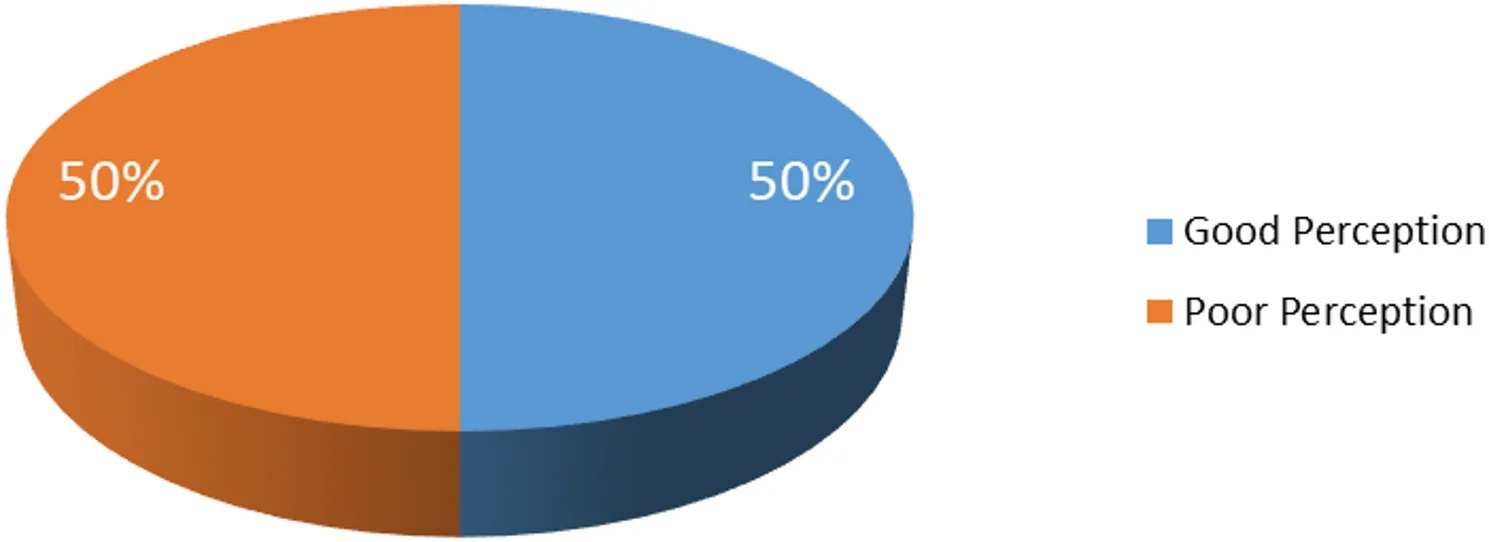
The level of patients’ perception towards nursing care among adult inpatients in comprehensive specialized hospitals of Northwest Ethiopia, 2023

Among the study participants, 301 participants (50%, 95% CI: 45.7–54.2) had a good perception of the nursing care they had received (Fig. 1).

### Factors related to patients’ perceptions towards nursing care

About fourteen variables were inserted into a binary logistic regression model for the bi–variable logistic regression analysis. Of those variables, nine variables, including the age of the patients, their marital status, their level of education, their occupation status, the residence where they lived, how long they were in the hospital, the admission ward they were admitted to, the type of ward service they received, and their prior history of admission, were significant at the significance level of 0.25.

The length of the hospital stay, previous history of hospitalization, and admission ward were found to be statistically significant predictors of patients’ perceptions of nursing care at p–values less than 0.05 in multivariable logistic regression analysis among sociodemographic and patient/admission–related factors included in this study.

This study’s regression analysis revealed that patients who had previously been hospitalized were 58% less likely than those who had never been admitted to perceive good nursing care (AOR = 0.42, 95% CI: 0.29–0.63; *P* < 0.001). In comparison to patients admitted to orthopedic wards, those admitted to medical wards were 57% less likely to perceive good nursing care (AOR = 0.43, 95% CI: 0.27–0.69; *P* = 0.001). Regarding the number of days spent in the hospital, patients who stayed for less than or equal to seven days were 1.5 times more likely to have good perceptions than those who stayed for greater than or equal to eight days (AOR = 1.50, 95% CI:1.03–2.20; *P* = 0.035).

### Qualitative study

The themes based on thematic analysis of 11 patients interviews guided by the described topics were Provision of Patient centered care, nurses provision of medical information, nurses timely provision of care, nurses attitudes towards patients, effectiveness of nurses on clinical skills, nurses way of communication to staffs, and nurses’ way of communication to patients.

### Provision of patient centered care

Based on the in-depth interview responses, the patients have different perceptions of the nursing care they received when they were hospitalized.

The majority of participants perceived negative patient-centered care; they feel they are not being listened, they feel their opinions was not considered and neglected by nurses when nurses are going to provide care.

It was described by 52 years-old- male participant as *“Due to current illness*,* I have been admitted to the hospital three times. However*,* some nurses in this hospital don’t seem to share my concerns. For example*,* most of the time nurse X followed me*,* but when I asked her to contact a doctor to transfer me to another nearby hospital*,* her response was*,* y*ou have to finish your course of treatment.” *[P-7]*.

On the other hand, some of the participants expressed a positive patient-centered approach to care that they have gained. The participants described episodes when they felt respected by nurses and willing to adjust their care to make them comfortable.

A 38-years-old female participant had said “*The nurses informed the doctors*,* and they changed my medication as soon as I told nurses*,* as the medication I had been taking wasn’t comfortable.”[P-2]*.

### Nurses provision of medical information

They believed that doctors recorded information in their medical records like; current medical status, brief information on the prognosis, dietary preferences, the side effects of treatments, information on patient discharge, and the outcome of hospitalization. Some patients felt that patients were not being informed about the current status of their illness or the reason for medication changes made. Additionally, patients have reported that they were not being given enough information about what to do and what to eat while they are admitted or after they have left the ward.

A 27 years old male participant stated that “*Even though Nurses were performing their tasks well*,* I wasn’t informed about the current status of my illness which might be recorded from my document.” [P-1].*

It also stated by a 36 years old male patient as *“When I was admitted to the hospital that referred me*,* the nursing staff briefed us about the disease*,* what we have to do*,* and our dietary preferences. But*,* since this admission*,* I haven’t seen them doing it in my ward.” [P–4]*.

In addition to that, a 40 years old female participant described it as; “*They do not give us information as why they are changing medications even when they are going to replace or add other medications.” [P-8]*.

In contrast, some patients perceived that the nurse was helpful in providing instructions regarding their care. They reported that the nurse provided information about a drug that can cause drowsiness and recommended rest while avoiding activities that could potentially cause harm.

A 38 years female participant stated as “*There was a medication that has drowsiness effect*,* due to this reason nurse advised me to have rest and to avoid potentially harmful activities while taking it.” [P-2]*.

### Nurses timely provision of care

The in-depth interview of the participant revealed that patients had varying levels of perceptions of the timeliness of care provided by nurses. Some patients had experienced delays in receiving timely care from nurses and they reported that the nurse doesn’t prioritize the patient’s urgent need for care. Patients who stayed hospitalized for a long time had been forgotten by nurses unless recalled by their nurses. Likewise some participants noticed that care given during daytime is preferable than nighttime due to potential delays in attention during the night.

The 27 years male participant responded as “*I remember that a nurse instructed my little brother to bring a condom catheter*,* and he promptly fulfilled the order. Even though I was soaked with urine they took beyond an hour to come.” [P-1]*.

In addition to this a 29 years male participant had also described as “Even if pain worsens at night, sometimes attention is delayed. Therefore care given at daytime is more preferable than nighttime.”[P-5].

It was also evidenced by the 40 years female participant “*They take care of us closely for a few days after surgery; however*,* after a period of time*,* it is an attendant who tells them when patients feel pain.”[P-8]*.

### Effectiveness of nurses on clinical skills

Based on the in-depth interview findings, the overall perception of the effectiveness of clinical skills by patients about nurses appeared to be somewhat varied. While some participants have praised the competency of the nurse who was able to secure the IV cannula after several attempts, others have reported negative experiences with the same procedure and other clinical skills such as wound care and catheterization. The participants have experienced unfavorable perceptions regarding the competency of nurses while inserting IV cannula and catheter, which have resulted in pain and discomfort. Furthermore, some patients felt that their wounds could have healed faster with better cleaning and care.

It was explained by 33 years male participant “*As an illustration*,* Nurse X repeatedly tried but failed to secure the intravenous line; nevertheless*,* another nurse quickly arrived and did it. Additionally*,* while catheterizing*,* he also made me feel pain.” [P-10]*.

### Nurses attitude towards patients

Majority of participants in the in-depth interview findings revealed that the nurses have a positive impact on the patients’ overall perception of the nursing care. The patients appreciated the nurses’ ability to feel their pain while administering medications and their consideration for privacy by making attendants stay outside during medical evaluations. Additionally, the fact that the nurses not only treated the patients with medications but also provided psychological therapy made them satisfied and increased their perception of the nursing profession as well. Furthermore, patients value the nurses’ dedication to preserving privacy even in cases where there were no curtains between patients. This led to an increase in trust toward nurses, as they seemed committed to ensuring that patients feel comfortable and safe.

The 20 years female participant stated it as “*while injecting medications they feel patients pain too and they make others to stay outside while we are going to have medical evaluation which made me even to have a good perception towards nursing profession. They also treated me not only by medications but also provided psychological therapy*,* which made me satisfied.”[P-9]*.

Unlike the perceived positive attitudes of nurses listed above, some patients had noticed that nurses had also negative caring behaviors, as evidenced by a 27 year old male participant as “*I have been admitted for a long period of time in this hospital*,* and it made them bored by me; that is why my questions were unconcerned. For example*,* I told them not to extend plaster while dressing my wounded part*,* but the response was not favorable to me.” [P–1]*.

This was also stated by 48 years male participant as *“I’ve been admitted to the hospital in the past*,* and the nurses were cleaning my wound while using a bedside screen and making the visitors and attendants leave the room. For current admission*,* while providing care*,* they make attendants stay outside*,* but there are a few gaps in the utilization of curtains to maintain our privacy.” [P–3]*.

### Nurse way of communication to staffs

Our participants had also clarified issues that constitute provision of quality nursing care. Effective communication and cooperation with colleagues during work shifts impact positively on the provision of optimal nursing care; hence, almost all patients realized that nurses’ provision of general information to doctors during round time and to their colleagues at duty shift time enhances patients’ health.

42 years male participant had described it as *“nurses provide the service in collaboration with their staffs*,* for instance*,* doctors prescribe our medications and promptly nurses deliver it to us.”[P-11]*.

Similarly, it was also explained by a 48year old male participant. *“It is their inter-professional communication which would make us to be treated well*,* and they would have knowledge about the medication that we take*,* we will be saved and they provide good service to us. If there is no communication among nurses how will patients be cured?” [P-3]*.

### Nurse way of communication to patient

The study participant considered that there was a communication gap between the nurses and patients. The participant agreed, as it is their culture to greet people, but some nurses don’t do it. This implies that some nurses were not showing basic communication gestures, which can make patients feel uncomfortable. On the other hand, the participant told that patients are always in tension when they are going to ask for help due to the fear of an unpleasant response from the nurses.

This is evidenced by a 29 years male participant *“If I see him once upon a time*,* I will greet him because it is our culture too*,* but here I can’t say all the nurses greet us by calling our names rather than few nurses.”[P-5]*.

Additionally, it was also stated by the quote of 52 years male participant *“However*,* we as a patient have needs*,* always we are in tension to ask for help even while seeking nursing care because we fear nurse’s response not to be unpleasant.” [P-7]*.

## Discussion

This study assessed patients’ perceptions of nursing care in comprehensive specialized hospitals of Northwest Ethiopia. The findings revealed that only half (50%; 95% CI: 45.7–54.2%) of the participants had a good perception of nursing care, indicating that a substantial proportion of patients still experience poor nursing care. Additionally, previous hospitalization history, admission ward, and length of hospital stay were identified as significant factors influencing patients’ perception.

The proportion of patients with a good perception in this study is slightly higher than findings from Eastern Ethiopia (45.2%) [11, 28]. And similar to findings from Western and Northern Ethiopia (49.3% and 50.3%, respectively) [1, 14, 29, 30]. This relative consistency across Ethiopian settings may suggest similarities in healthcare system structure, nursing workforce challenges, patient load, and resource limitations. However, minor variations could be related to differences in study settings, sample characteristics, and measurement tools. Similarly, the current finding is consistent with a study conducted in Iran (51.6%) [19] but higher than Nigeria (18.5%) [13]. The significantly lower result in Nigeria may be linked to differences in healthcare infrastructure, patient expectations, and, in particular, the use of a different assessment tool, the modified Consumer Quality Index, which can influence how perception is measured and interpreted.

However, this study found a lower proportion of patients with a good perception of nursing care compared to studies conducted in countries such as Spain (94%), Indonesia (82.19%), Nepal (63.6%), Pakistan (57%), and Sri Lanka (70%) [16, 18, 19, 25, 26]. This difference might be due to multiple factors. First, variations in healthcare system capacity, such as nurse–to–patient ratios, availability of medical resources, and organizational support, may lead to better patient experiences in higher–performing systems. Also, sociocultural differences can influence patients’ expectations and perceptions of care. Furthermore, methodological differences, particularly in the tools used to measure patient perception, make direct comparisons across studies difficult. Therefore, the observed gap may reflect not only differences in the quality of care but also differences in context and measurement methods. Regarding the associated factors, a previous history of hospitalization was significantly associated with patients’ perception of nursing care. Here, it was found that patients who had previously been hospitalized were 58% less likely to perceive good nursing care than patients who had no prior hospitalizations. This result was consistent with research done in selected public hospitals of Eastern Ethiopia [31]. Besides this, it was confirmed by the in-depth interview of the participant as:

When I was admitted to the hospital that referred me, the nursing staff briefed us about the disease, what we have to do, and our dietary preferences. But, since this admission, I haven’t seen them doing it in my ward. [P–4]

And it was also supported by another participant’s quote as “I’ve been admitted to the hospital in the past, and the nurses were cleaning my wound while using a bedside screen and making the visitors and attendants leave the room. For current admission, while providing care, they make attendants stay outside, but there are a few gaps in the utilization of curtains to maintain our privacy.” [P–3].

Compared to patients admitted to orthopedic wards, patients in medical wards were 57% less likely to perceive good nursing care. This result was in line with the research conducted in eastern Ethiopia [28]. This might be because most of the time medical ward patients have more serious illnesses, poorer prognoses, and are subjected to stressful and uncomfortable conditions; as a result, these things may have influenced their perception level and made it low.

Patients who stayed less than or equal to seven days had a 1.5 times higher likelihood of having a positive perception than those who were admitted for at least eight days. Evidence for this finding was revealed by a study conducted in western and eastern Ethiopia [1, 28]. It might be because patients who stay in hospitals for a long period of time have opportunities to observe nurses’ caring behaviors, which enhances expectations for nursing care, or it might be because patients who stay in hospitals for longer periods of time may have more hospital–acquired infections that weaken patients and cause their expectations to rise. This finding was supported by the qualitative result of the participant’s quote, stated as follows:

I have been admitted for a long period of time in this hospital, and it made them bored by me; that is why my questions were unconcerned. For example, I told them not to extend plaster while dressing my wounded part, but the response was not favorable to me. [P–1]

This finding was also supported by the quotes of a 40–year–old female participant: “*They take care of us closely for a few days after surgery; however*,* after a period of time*,* it is an attendant who recalls them when patients feel pain.” [P–8]*.

The results of this investigation have significant clinical and public health implications. Ensuring privacy and dignity during treatment, improving communication between nurses and patients, and enhancing patient–centered care approaches are all necessary to improve patients’ perceptions of nursing care. Patients in medical wards and those with extended hospital stays should receive special care. Healthcare facilities should also put in place procedures for ongoing monitoring and assessment in order to evaluate patient satisfaction and direct efforts to enhance quality. Improving treatment compliance, patient satisfaction, and general healthcare results all depend on addressing these problems.

### Limitations of the study

This study has some limitations that should be considered when interpreting the findings. The cross–sectional design limits the ability to establish causal relationships between identified factors and patients’ perception of nursing care. In addition, recall bias may have occurred, as participants were asked to reflect on their experiences during hospitalization. Furthermore, although the study used a mixed–methods approach, the findings may not be fully generalizable to other healthcare settings in Ethiopia beyond the selected comprehensive specialized hospitals.

## Conclusion

The findings of this study specifically demonstrated that a significant proportion of patients reported poor perceptions of nursing care, highlighting a critical gap in the quality of care delivery. Patients’ perceptions were significantly influenced by contextual and experiential factors, including admission ward, prior hospitalization history, and length of hospital stay. These results indicate the need for targeted, evidence-based interventions related to specific clinical settings and patient experiences. Strengthening patient-centered care practices, improving communication, and addressing ward-specific challenges are essential to enhancing patients’ perceptions of nursing care. Therefore, healthcare institutions and nursing professionals must prioritize strategic actions and continuous quality improvement initiatives to ensure more positive patient experiences and optimal care outcomes.

## Supplementary Information

Below is the link to the electronic supplementary material.

Supplementary Material 1

## Data Availability

All necessary data are available from the corresponding author at any time.

## Abbreviations

AOR: Adjusted odds ratio
CBHI: Community Based Health Insurance
BDU CMHS: Bahir Dar University College of Medicine and Health
CI: Confidence interval
COR: Crud odds ratio
DMCSH: Debremarkos comprehensive specialized hospital
DTCSH: Debre Tabor Comprehensive Specialized
FHCSH: Felege Hiwot Comprehensive Specialized Hospital
IPD: Inpatient department
SPSS: Statistical Package for Social Science
TGCSH: Tibebe Ghion Comprehensive Specialized Hospital
UOGCSH: University of Gondar Comprehensive Specialized Hospital
