# Supplementary material for: Patients’ perception towards nursing care and its associated factors in adult inpatient departments in comprehensive specialized hospitals of Northwest Ethiopia, 2023: a mixed methods study
Source: BMC Nurs. 2026 Jul 3;25:941. doi: 10.1186/s12912-026-04988-z (PMC13647653; doi:10.1186/s12912-026-04988-z)
Supplement: Supplementary file 1 [file 12912_2026_4988_MOESM1_ESM.docx]

**Supplementary figures**

Figure 1: Schematic representation of the sampling procedure for the study on patient’s perception of nursing care in comprehensive specialized hospitals of Northwest Ethiopia, 2023.

**Supplementary tables**

Table 1: Factors associated with patient’s perception towards nursing care at comprehensive specialized hospitals of Northwest Ethiopia, 2023

| Variable | Category | Patients’ perception | | COR (95%CI) | AOR(95%CI) |
| --- | --- | --- | --- | --- | --- |
|  |  | Good No. (%) | Poor No. (%) |  |  |
| Age of patients | 18-30 | 119(39.5) | 101(33.6) | 2.108(1.387-3.205) | 1.364(0.719-2.589) |
|  | 31-40 | 63(20.9) | 47(15.6) | 2.399(1.458-3.946) | 1.524(0.847-2.741) |
|  | 41-50 | 62(20.6) | 51(16.9) | 2.175(1.330-3.559) | 1.585(.899-2.792) |
|  | >50 | 57(18.9) | 102(33.9) | 1 | 1 |
| Marital status | Single | 88(29.2) | 77(25.6) | 1 | 1 |
|  | Married | 162(53.8) | 173(57.5) | 0.819(0.564-1.190) | 1.114(0.666-1.863) |
|  | Separated | 15(5) | 9(3) | 1.458(0.604-3.520) | 1.469(0.523-4.128) |
|  | Divorced | 26(8.6) | 13(4.3) | 1.750(0.841-3.641) | 2.306(0.981-5.418) |
|  | Widowed | 10(3.3) | 29(9.6) | 0.302(0.138-0.659) | 0.956(0.348-2.625) |
| Educational status | Unable to read and write | 85(28.2) | 115(38.2) | 0.535(0.317-0.902) | 0.549(0.237-1.237) |
|  | Able to read and write | 38(12.6) | 54(17.9) | 0.509(0.278-0.933) | 0.548(0.235-1.278) |
|  | Primary school(1-8) | 72(23.9) | 46(15.3) | 1.132(0.637-2.014) | 1.221(0.578-2.578) |
|  | Secondary school(9-12) | 59(19.6) | 52(17.3) | 0.821(0.461-1.463) | 1.076(0.519-2.234) |
|  | College diploma and above | 47(15.6) | 34(11.3) | 1 | 1 |
| Occupation | Government employee | 51(16.9) | 36(12) | 3.010(1.173-7.725) | 1.517(0.462-4.978) |
|  | Merchant | 46(15.3) | 46(15.3) | 2.125(0.835-5.410) | 1.388(0.478-4.031) |
|  | Farmer | 111(36.9) | 106(35.2) | 2.225(0.922-5.373) | 2.652(0.925-7.608) |
|  | House wife | 29(9.6) | 36(12) | 1.712(0.647-4.526) | 1.157(0.382-3.507) |
|  | Student | 32(10.6) | 32(10.6) | 2.125(0.803-5.621) | 1.181(0.381-3.655) |
|  | Private employee | 24(8) | 28(9.3) | 1.821(0.669-4.960) | 1.087(0.348-3.394) |
|  | Others | 8(2.7) | 17(5.6) | 1 | 1 |
| Admission Ward | Medical | 92(30.6) | 155(51.5) | 0.4070.261-0.633) | 0.430(0.266-0.694)** |
|  | Surgical | 136(45.2) | 96(31.9) | 0.970(0.622-1.514) | 1.129(0.698-1.826) |
|  | Orthopedic | 73(24.3) | 50(16.6) | 1 | 1 |
| Length of hospital stay | >=7 | 86(28.6) | 108(35.9) | 1.399(0.992-1.972) | 1.504(1.030-2.197)* |
|  | >=8 | 215(71.4) | 193(64.1) | 1 | 1 |
| service type | Free | 26(8.6) | 23(7.6) | 0.958(0.503-1.821) | 1.1100(.481-2.560) |
|  | CBHI | 190(63.1) | 206(68.4) | 0.781(0.539-1.132) | 1.011(0.635-1.610) |
|  | Payment | 85(28.2) | 72(23.9) | 1 | 1 |
| Previous history of admission | Yes | 69(22.9) | 127(42.2) | 0.407(0.286-0.580) | 0.424(0.285-0.630)** |
|  | No | 232(77.1) | 174(57.8) | 1 | 1 |
| Residence | Rural | 156(51.8) | 177(58.8) | 0.754(0.546-1.040) | 0.648(0.391-1.074) |
|  | Urban | 145(48.2) | 124(41.2) | 1 | 1 |

“⁎”- indicates statically significant (p <0.05) whereas “⁎⁎” - indicates statically highly significant (p <0.01), AOR =adjusted odd ratio, COR=crude odd ratio, CI=confidence interval & Reference=1
